# Suspension feeding in Copepoda (Crustacea) – a numerical model of setae acting in concert

**DOI:** 10.3762/bjnano.14.50

**Published:** 2023-05-17

**Authors:** Alexander E Filippov, Wencke Krings, Stanislav N Gorb

**Affiliations:** 1 Department of Functional Morphology and Biomechanics, Zoological Institute, Christian-Albrechts-Universität zu Kiel, Am Botanischen Garten 1–9, 24118 Kiel, Germany; 2 Donetsk Institute for Physics and Engineering, National Academy of Sciences of Ukraine, 83114 Donetsk, Ukraine; 3 Department of Behavioral Biology, Institute of Cell and Systems Biology of Animals, Universität Hamburg, Martin-Luther-King-Platz 3, 20146 Hamburg, Germany; 4 Department of Mammalogy and Paleoanthropology, Leibniz Institute for the Analysis of Biodiversity Change, Martin-Luther-King-Platz 3, 20146 Hamburg, Germany; 5 Department of Cariology, Endodontology and Periodontology, Universität Leipzig, Liebigstraße 12, 04103 Leipzig, Germany

**Keywords:** adhesion, confocal laser scanning microscopy (CLSM), feeding efficiency, feeding structures, mechanical properties

## Abstract

Suspension feeding via setae collecting particles is common within Crustacea. Even though the mechanisms behind it and the structures themselves have been studied for decades, the interplay between the different setae types and the parameters contributing to their particle collecting capacities remain partly enigmatic. Here, we provide a numerical modeling approach to understand the relationship among the mechanical property gradients, the mechanical behavior and the adhesion of setae, and the feeding efficiency of the system. In this context, we set-up a simple dynamic numerical model that takes all of these parameters into account and describes the interaction with food particles and their delivery into the mouth opening. By altering the parameters, it was unraveled that the system performs best when the long and short setae have different mechanical properties and different degrees of adhesion since the long setae generate the feeding current and the short ones establish the contact with the particle. This protocol can be applied to any system in the future as the parameters (i.e., properties and arrangement of particles and setae) can be easily altered. This will shed light on the biomechanical adaptations of these structures to suspension feeding and provide inspiration for biomimetics in the field of filtration technologies.

## Introduction

Particle capture mechanisms are common in a huge variety of aquatic animals, such as polychaetes, bryozoans, bivalves, sponges, echinoderms, cnidarians, or crustaceans [1–7]. Even though living conditions and bauplans differ between suspension feeders, there are two main mechanisms for particle collection from the water body (for in-depth reviews on suspension feeding, see [8–11]). The first one can be described as filtering or sieving with, for example, setae, cilia, or mucous nets, and is present in form of passive or active suspension feeding. Passive feeders rely on external water currents that bring food particles to the filtering structures and active feeders create a feeding flow by pumping systems. The second mechanism involves structures manipulating the water flow (e.g., setae and tentacles) that redirect the food particles and lead them to specialized structures that contact and capture them. A good example for the latter mechanism are the filtering setae of crustaceans (for in-depth reviews, see [12–13]). Even though most crustaceans are primarily raptorial, suspension feeding plays an important role. In general, multiple pairs of appendages generate the feeding current, and the particles are captured by plumate “filter setae”, which cover the trunk and head appendages. These setae have to establish contact with the particles by inertial impaction and capture and transport them to the mouth opening [14–18].

These interactions (i.e., making contact with and handling of or manipulating particles) were previously documented in detail through observation under a binocular microscope [19–28]. In this context, setae morphology and mesh size of the filtering structure and the surface chemistry and forces (e.g., van der Waals forces) of feeding structures and particles are of high importance, especially when the particles are of smaller diameter than the meshes of the sieve [14,29–36]. Additionally, the mechanical property gradients of the setae, with soft bases or soft tips, seem to play a role [25,37–38].

**Figure 1:**
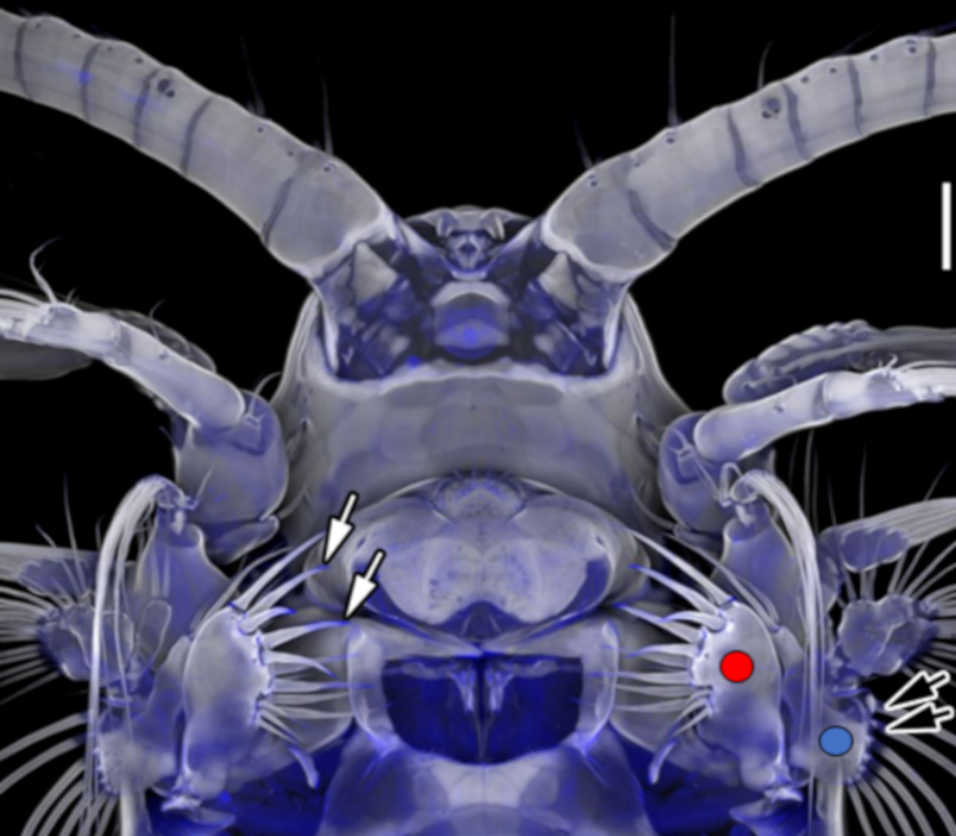
Confocal laser scanning micrograph (maximum intensity projection) showing the exoskeleton of a female copepod crustacean *Centropages hamatus* in ventral view. The black arrows highlight the outer long setae with resilin occurring at their bases. The white arrows indicate the setae with resilin occurring at their tips. The red circle highlights maxilla 1 and the blue one maxilla 2. Scale bar on the right side = 50 µm. Figure 1 was adapted (by adding arrows and circles) from [57], J. Michels, “Confocal laser scanning microscopy – detailed three-dimensional morphological imaging of marine organisms”, Imaging Marine Life, with permission from John Wiley and Sons. Copyright © 2014 Wiley-VCH Verlag GmbH & Co. KGaA. This content is not subject to CC BY 4.0.

All of the abovementioned parameters influence the capability of the setae to capture and transport the particles, but to which extent is unknown since these parameters cannot be manipulated in living organisms. To test how the feeding efficiency depends on the mechanical property gradients and the adhesion forces of the setae, we here present a numerical model that simulates the interplay between setae during suspension feeding. In the past, numerical simulations were used to study the detection of prey, mates, or predators and the feeding current generation by limb motion [28,39–41]. However, mechanical property gradients and adhesion of setae were previously not addressed. As model organism we chose the copepod *Centropages hamatus* (Lilljeborg, 1853). This species belongs to the Calanoida, where filter feeding is the derived condition [19–20,29,42–54]. For this species (Figure 1), previous confocal laser scanning microscopy (CLSM) studies on the cuticle’s mechanical properties revealed that the setae on maxillae 1 (long setae) and 2 (short setae) possess very soft bases full of the elastic protein resilin [55–57]. Additionally, the tips of the short setae on maxilla 2 exhibited a blue autofluorescence signal, which strongly indicated that these tips are also rather soft and flexible, similar to attachment hairs in insects showing high adhesion at the tips [58]; for in-depth reviews, see [59–61]. In contrast, the tips of the long setae did not emit blue signals.

The simulation presented here takes into account the actual physical processes of the water body (including the interplay between particles). Two types of setae (long and short ones) were arranged on crests, similar to the real situation in copepods, and their parameters (adhesion and mechanical property gradients) were altered. The model produces data on the effectivity of particle collection, the particle motion patterns, and the transfer of particles to the mouth opening. It clearly depicts that short and long setae are more effective when they work in concert and have different mechanical properties and different adhesion forces. This study is rather a protocol for carrying out more extensive numerical modeling in the future as the model can be easily computed with MatLab, and the parameters of the model (e.g., the size of the food particles and the quantity and mechanical properties of setae) can be adjusted to specific systems or problems. This model shall serve as a basis to unravel the interplay between the feeding structures of suspension feeders, the preferred food, and the gathering performance.

Additionally, it could open new avenues in the development of new filtration technologies (e.g., mucus-like filter media or bioinspired membranes) that use adhesive forces to retain particles. In contrast to organisms, which collect particles at the nano- to millimeter scale, most industrial cross-flow filtration systems can capture material only in more limited size ranges, highlighting the necessity to investigate particle retention in biological systems.

## Results and Discussion

### Numerical simulations

In this study, we restricted ourselves to a few biologically important questions:

1. Is there a difference in feeding performance between a system possessing only short setae near the mouth opening and a system with both types of setae, long and short ones?
2. Which mechanical properties (flexible or stiff) of the setae segments facilitate the feeding of particles?
3. How does the feeding efficiency change when the setae tips have a high adhesion?
4. How does the feeding efficiency change when the basic segments of each setae are more flexible and allow for a higher bending amplitude?

**Figure 2:**
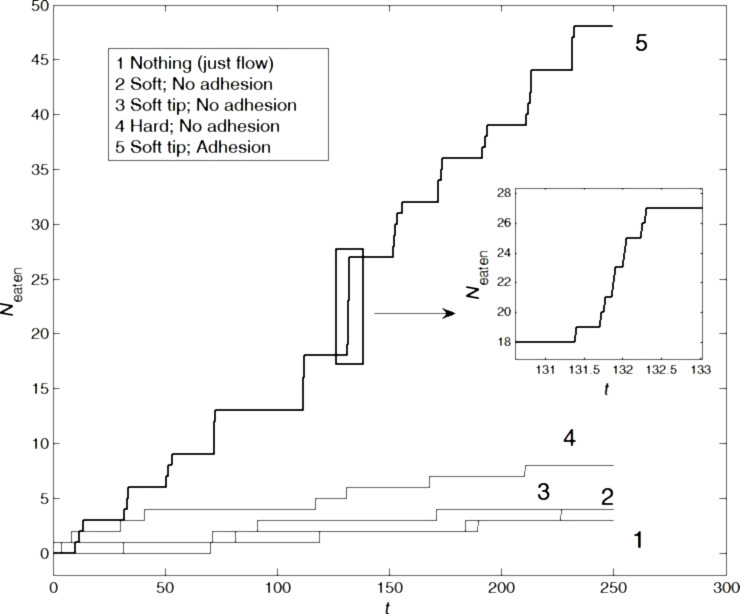
Number of eaten particles as a function of the time for systems containing only short setae (lines 2–5). Line 2 corresponds to a system with soft setae without high adhesion at the tips, line 3 to hard setae with soft tips and without high adhesion at the tips, line 4 to hard setae without high adhesion at the tips, and line 5 to hard setae with soft tips and high adhesion at the tips. Line 1 corresponds to the reference system, which does not contain setae at all (food particles are transported into the mouth by water motion). The insert depicts the fine structure of one typical big step corresponding to an avalanche of eaten particles during a relatively short time interval. The characteristic time intervals between the avalanches correlate with the periodic oscillations (rotations) of the system. The bold curve highlights the optimal configuration (hard setae with soft tips and high adhesion at the tips).

**Figure 3:**
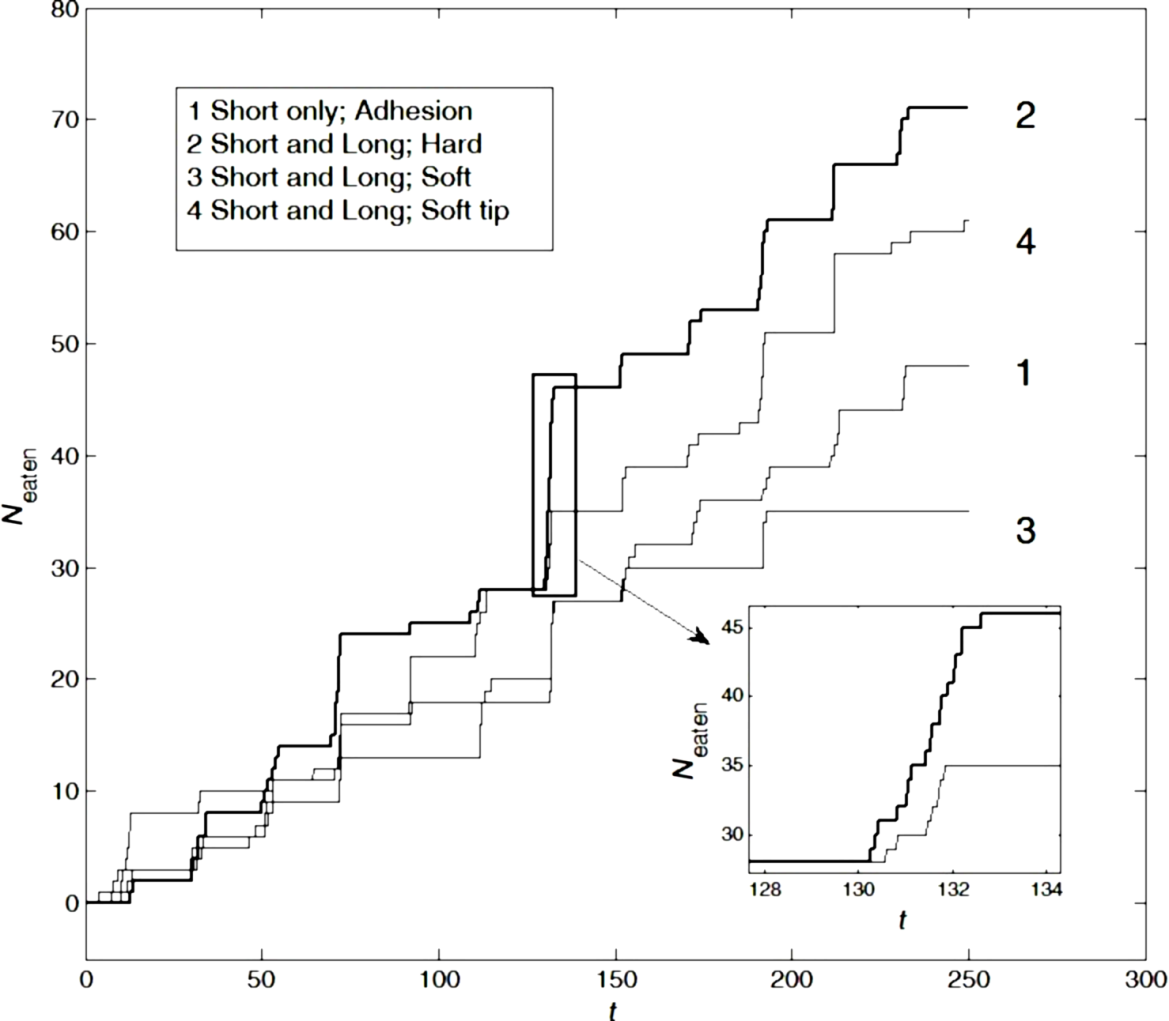
The same as Figure 2, but for systems containing both long and short setae. Line 1 corresponds to the optimal configuration of short setae, taken from the Figure 2 (hard short setae with soft tips and high adhesion at the tips; line 5). For the long setae, no adhesion was chosen. Lines 2–4 correspond to configurations in which the short setae had the same optimal properties and the properties of the long setae were varied: Line 2 depicts the quantity of ingested particles by hard long setae without adhesion at their tips, line 3 represents soft long setae with high adhesion at their tips, and line 4 represents hard setae with soft tips without adhesion at their tips. The insert shows a minor time interval with visually resolved avalanches. Different time intervals between larger steps with different heights correspond to random mutual correlations in motion of the short and long setae. The bold line 2 highlights the optimal configuration with hard long setae without adhesion at their tips.

**Figure 4:**
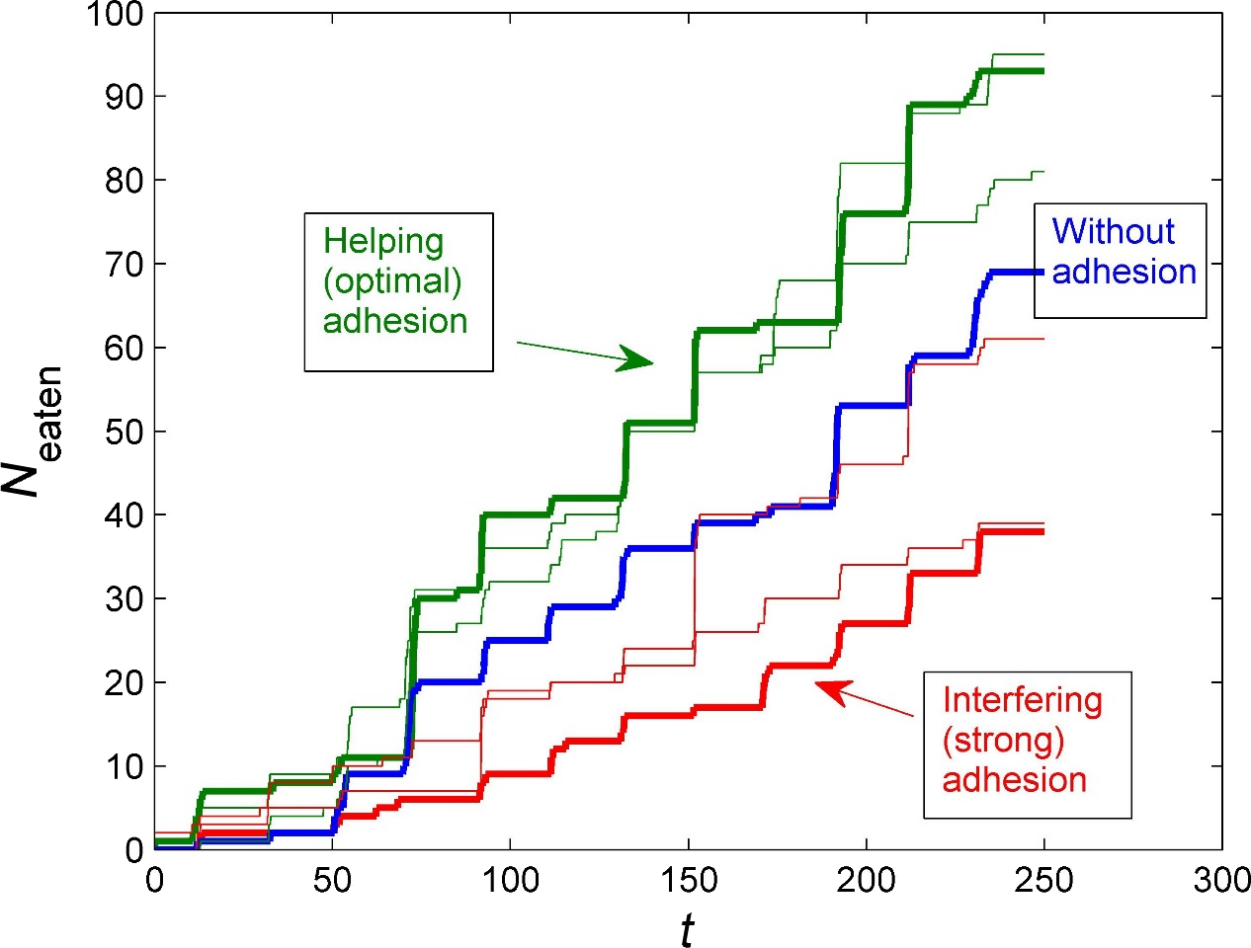
Number of eaten particles as a function of the time for the systems containing both short and long setae, where nonzero adhesion of the long setae also exists. All other parameters are the same as those optimized for the system without adhesion of the long setae. The blue line corresponds to a system with setae without adhesion at their tips, the red line to setae with strong adhesion at their tips, and the green line to setae with intermediate adhesion at the tips. The green curve represents the optimal configuration.

**Figure 5:**
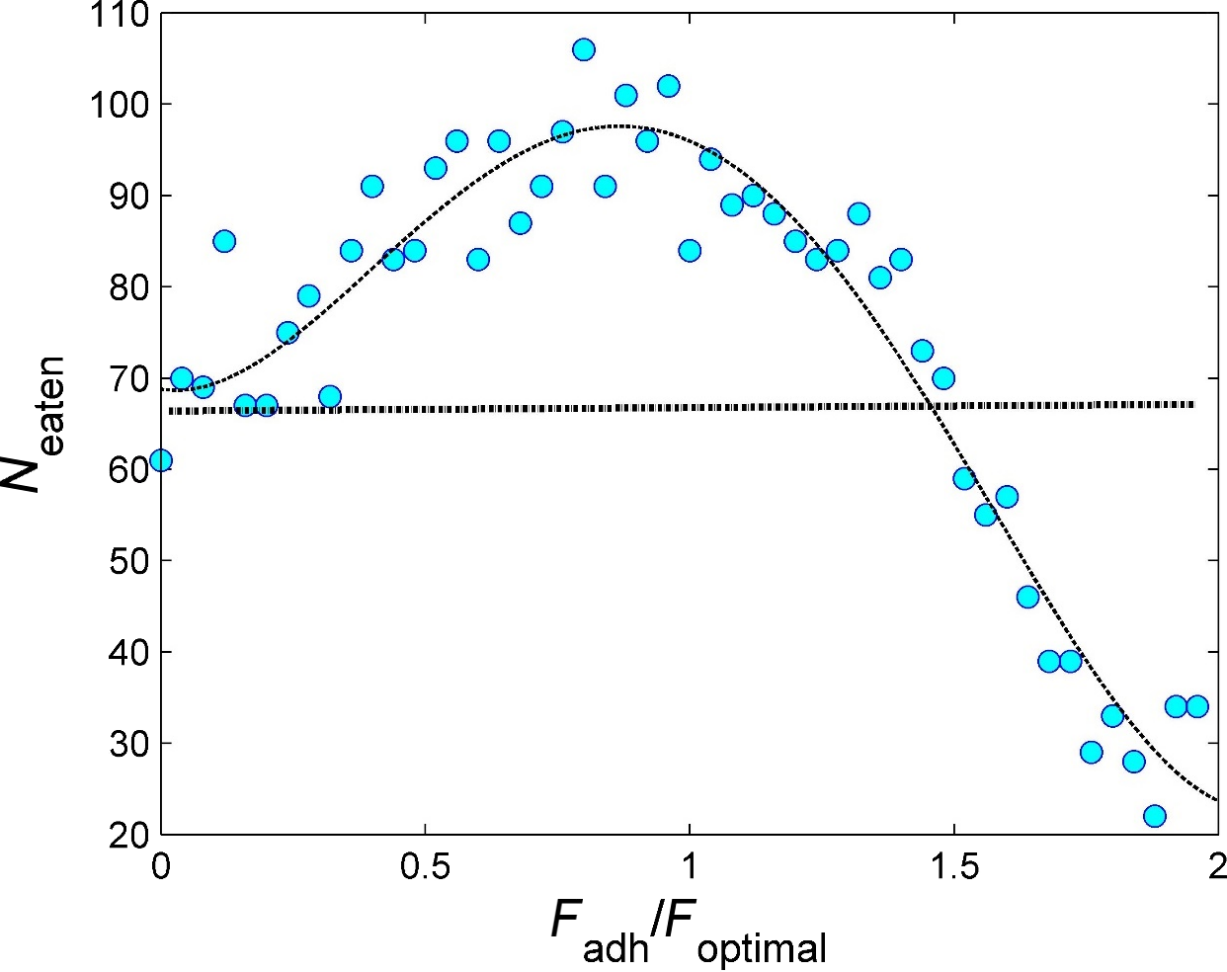
Number of eaten particles for different degrees of adhesion of the long setae tips. Multiple experiments were performed. Each blue circle here corresponds to the final number *N*_eaten_(*t*) obtained at the end *t* = 250 of a long-time simulation run, analogous to the results in the previous figure at random initial configuration of the food particles and varied step-by-step adhesion force. It is obvious that the highest number of particles is eaten at intermediate adhesion. When the adhesion is too strong, the food particles cannot be transported into the mouth opening. If, and to which extent, long setae have adhesion on their tips in real copepods awaits further investigations.

To elucidate this, we performed a set of numerical simulations with different configurations of the setae, the segments’ elasticity, and the adhesion of the segments. The relationship between different variants of elasticity for a system composed of only short setae, as well as for a system containing short and long setae, and the number of eaten particles is summarized below in Figures 2–5.

Figure 2 represents the time dependencies of *N*_eaten_(*t*) for four different variants of short setae. Line 2 represents soft setae without high adhesion at the tips, line 3 represents hard setae with soft tips and without high adhesion at the tips, line 4 represents hard setae without high adhesion at the tips, and line 5 represents hard setae with soft tips and high adhesion at the tips. The latter configuration led to the consumption of the highest number of particles.

These variants were numbered correspondingly in Figure 2. For comparison, we also included a curve (line 1 in Figure 2) that depicts the number of eaten particles *N*_eaten_(*t*) in a system without setae. Here, only the flow of water randomly transports particles to the mouth opening and causes a slow accumulation of *N*_eaten_(*t*).

It is important to note that the large steps in the curves are not caused by the accuracy of the calculations, but appear only as an “optical illusion” due to the presentation of the figure in limited size. In fact, each step is one of the consumption avalanches that appear quasi-periodically during the simulation. At appropriate magnification, every large step of the curve has a fine structure with plenty of small steps. Due to the small accumulation window (coinciding with the elementary time interval of the actual calculation), every such step can be resolved down to the independent consumption of sole particles. This fine structure is illustrated for one of the typical avalanches in Figure 2. Most particles are eaten when the short setae are hard and contain soft tips with high adhesion at the tips. Such properties of short setae were previously determined by using CLSM with real specimens [55–57] and are here shown to increase the feeding efficiency.

Analogous dependencies *N*_eaten_(*t*) are plotted in Figure 3 for the system with both short and long setae. For this scenario, we chose the optimal case from the previous scenarios (hard short setae with soft tips and high adhesion at the tips, highlighted with a bold line in Figure 2) as a reference curve (number 1) in Figure 3. For the long setae we have chosen a setup without adhesion at their tips. We simulated the following scenarios for long setae: Line 2 represents hard setae (this configuration led to the consumption of most particles), line 3 represents soft setae, and line 4 represents hard setae with soft tips. It is obvious that line 2 corresponds to the maximal value of *N*_eaten_(*t*). As above, a bold line highlights the optimal case, and some typical avalanches are magnified in Figure 3.

The above CLSM image reveals that the long setae do not exhibit blue autofluorescence, and that there is most likely no adhesion on the tips [55–57]. However, to test if adhesion on these setae would influence the feeding capacity, we varied the degree of adhesion. Figure 4 shows the time dependencies of *N*_eaten_(*t*) for three different variants of the long setae, namely (1) without adhesion at the tips, (2) with strong adhesion at the tips, and (3) with intermediate adhesion at the tips. This configuration leads to the consumption of more particles, because, due to adhesion, food particles follow the setae and come in the vicinity of the mouth, where short setae collect them and transport them into the goal. However, when the adhesion is too strong, the food particles continue to follow the setae, even after their appearance in the vicinity of the short setae, and almost never enter the mouth.

We additionally altered the degree of adhesion in more detail and performed multiple experiments (Figure 4 and Figure 5). From these experiments, it becomes obvious that there is an optimal degree of adhesion for the long setae, which supports the system. Whether adhesion is present in real structures should be investigated in the future by using either high-resolution CLSM imaging or atomic force microscopy.

As it was visualized by CLSM [55–57], the basal parts of some short and long setae appear to be relatively soft and seem to contain resilin or other proteins. This should influence the mobility of the rotating setae. To account for this in the numerical simulations, one can integrate an angle to which every seta can rotate toward the mouth, ϕ_min_.

**Figure 6:**
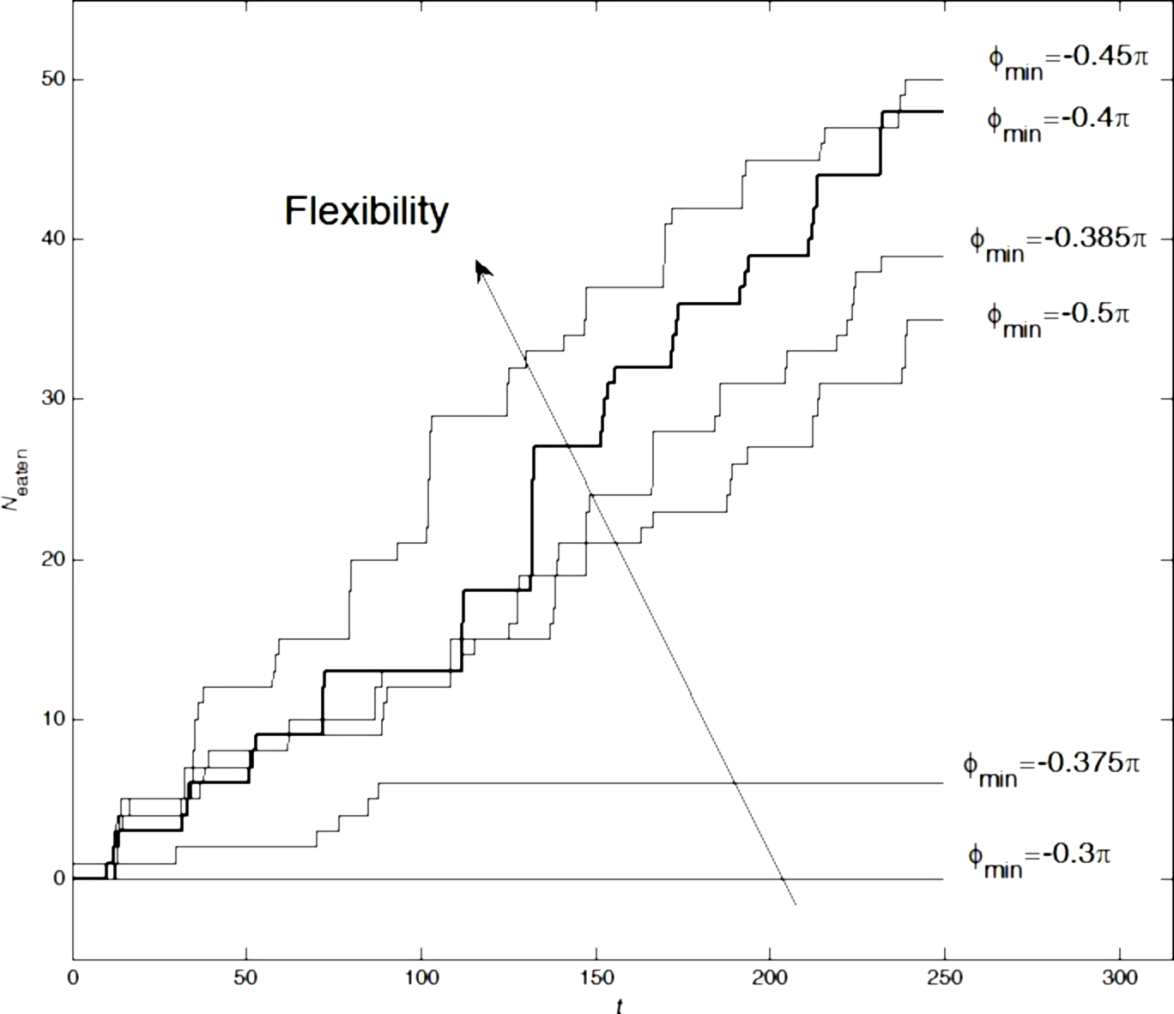
Time dependencies of *N*_eaten_ at different angles of rotation for the basic segments of the short setae. The threshold angle, ϕ_min_ = −0.375π, at which the system stopped delivering particles into the mouth opening is easily identified. The optimum ϕ_min_ = −0.4π is highlighted with the bold line and was used for all previous simulations.

For the first simulations with ϕ_min_, we excluded the long setae and only simulated the system with short setae (with the optimal configuration, i.e., with soft adhesive tips). The results of this simulation are summarized in Figure 6.

As seen directly from the plots, the well-pronounced threshold angle is ϕ_min_ = −0.375π. Below this threshold, there is practically zero food consumption. Paradoxically, the quantity of ingested particles is even smaller than in the reference system without setae, that is, with only water flow. Visual observation of the behavior in the simulations showed that when the absolute value of ϕ_min_ was smaller than the critical value, the short setae caught surrounding particles and permanently moved them back and forth (“screening”). As result, they practically blocked the mouth entrance. It was also found, that the particular angle ϕ_min_ = −0.4π is very close to the optimum. This value was actually used for all the simulations presented in the previous Figures 2–5 and to record the movie in the Supporting Information File 1.

**Figure 7:**
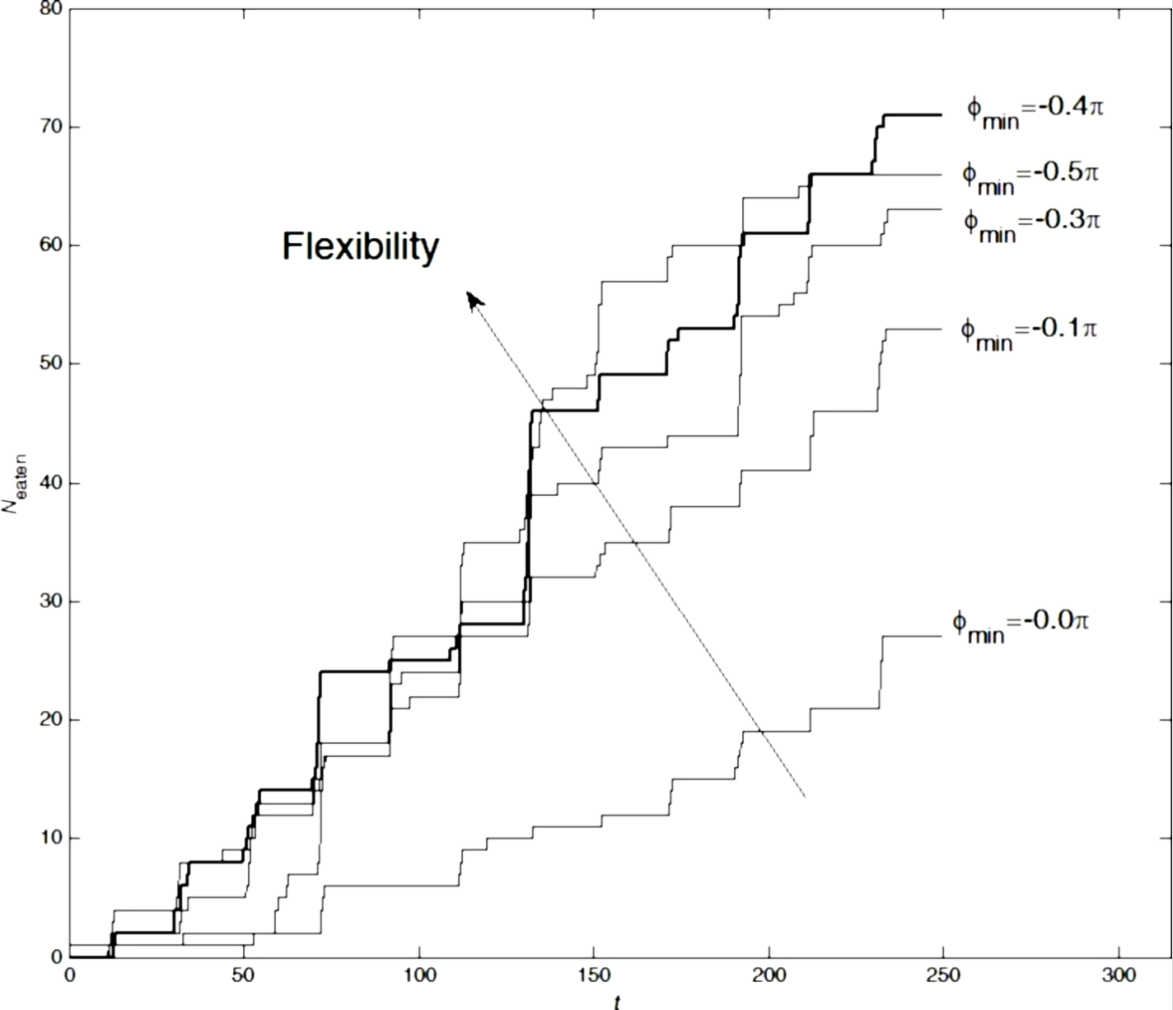
The same as in Figure 6 for the system containing both long and short setae. The short setae had the optimal fixed angle of rotation ϕ_min_ = −0.4π; the angle of rotation for the long setae was varied. The bold line highlights the curve corresponding to the optimal angle.

The same calculations were done for a system containing short and long setae. The results are presented in Figure 7. It is important to note that the angle ϕ_min_ = −0.5π (here the basic segments run parallel to the surface *z* = 0) yields worse results than the optimal angle ϕ_min_ = −0.4π.

**Figure 8:**
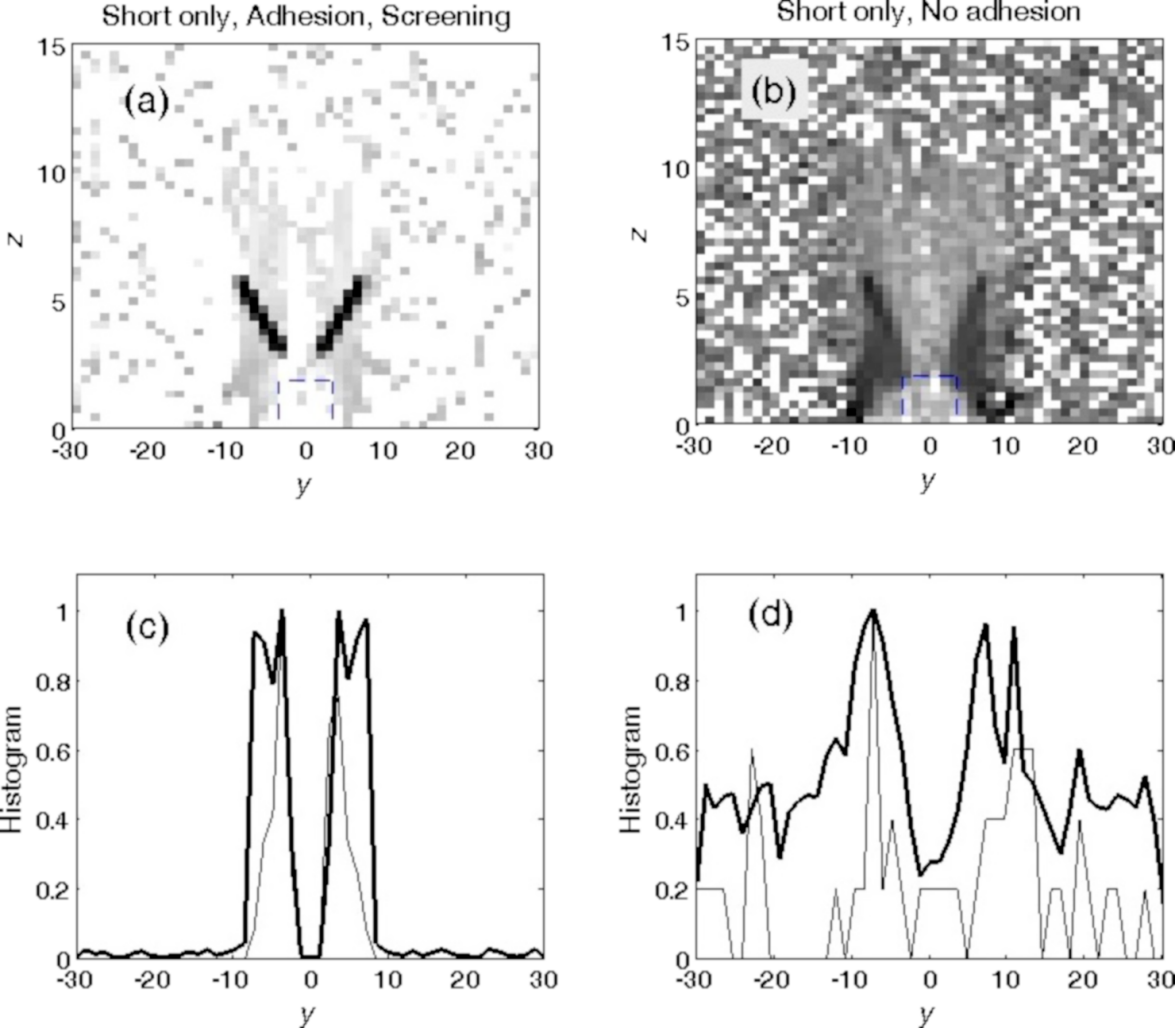
Density distributions obtained from long-time simulation runs in the (*y*,*z*) plane shown by grayscale maps, which were normalized to the density maximums, and the corresponding histograms integrated additionally over the *z* axis. Darker colors correspond to a higher density of particles. The thin curves represent instant histograms, and the bold curves the number of particles averaged over time. Panels (a, c) and (b, d) illustrate the particle distributions for short setae with and without adhesion, respectively. “Screening” in (a, c) means that the rotation angle was smaller than the critical angle. This means that the setae practically did not allow particles to enter the mouth.

**Figure 9:**
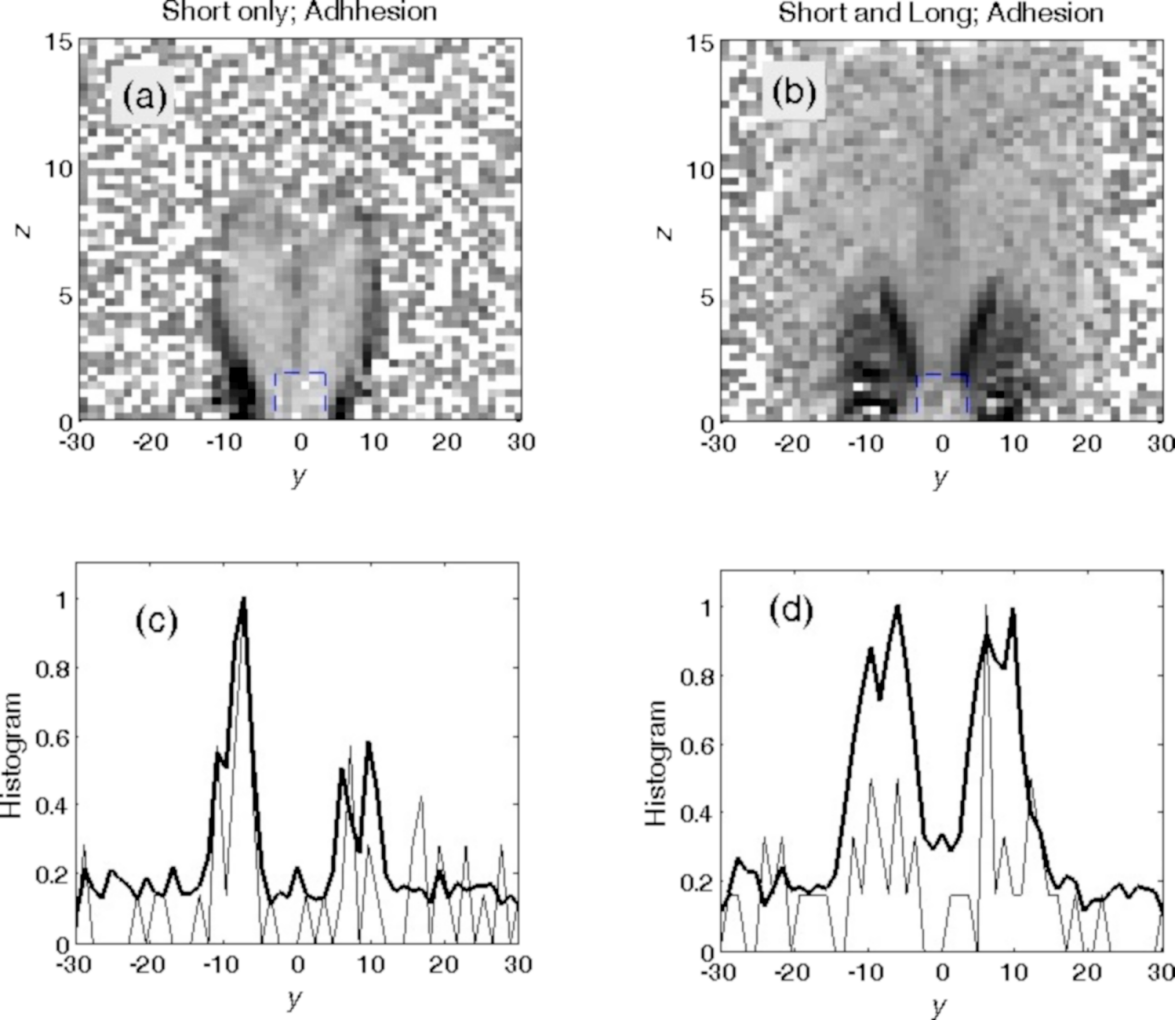
The same as in Figure 8 for two optimal configurations. (a, c) Only short setae (hard setae with soft tips and high adhesion at the tips) and (b, d) long setae (hard with no adhesion at their tips) and short setae (hard with soft tips and high adhesion at the tips). Smooth gray areas correspond to regions with good statistics, where plenty of the particles were accumulated and quickly moved into the mouth. Black spots on the left and right sides of the mouth show the places where particles accumulate over time but cannot enter the mouth. The difference in accumulation for these two cases can be directly seen. The results in (b, d) can be identified as the optimal configuration to collect particles from the surrounding water.

The dynamic behavior of the systems with different parameters can be presented in a static form by density distributions projected on the (*y*,*z*) plane and histograms of the distributions along the *y* axis accumulated during long-time simulation runs. Figure 8 and Figure 9 present the results of such an accumulation for four cases with extremely different kinds of behavior. Darker colors in the grayscale maps correspond to a higher food particle density. Thin curves represent instant snapshots of the particles’ location, and the bold curves represent the number of particles averaged over time, respectively.

The panels in Figure 8 illustrate the particle distributions for short setae with (Figure 8a,c) and without (Figure 8b,d) adhesion. The first pair of the panels (Figure 8a,c) clearly demonstrates what happens during the “screening” described above, which appears when the rotation angle is smaller than the critical angle and the setae do not allow particles to enter the mouth opening. One can see very dark regions in the map where particles spent most of the time, following the periodic rotation of the setae without entering the mouth opening. The corresponding histogram integrated over time and the vertical direction confirmed the particle localization in a small region. It even reproduced well-pronounced maximums near so-called “stopping points”, where the rotation changes direction. In these places, particles, due to inertia, left for a short time the close proximity of the setae tips, but were very soon attracted to the setae again. The second pair of the panels (Figure 8b,d) shows that particles are much more widely dispersed when the system lacks adhesion. In this case, particles enter the mouth from time to time, but many of the trajectories still lead into the “wrong direction”. As a result, the particles periodically repeat many “parasitic” oscillations before they finally enter the mouth.

The plots in Figure 9 reproduce the results of two almost optimal configurations found above for a system containing only short setae (with adhesion at their tips) and for the system containing long and short setae (short setae with adhesion at their tips and hard long setae without adhesion on their tips; rotation angle ϕ_min_ = −0.4π). They are depicted in Figure 9a,c and Figure 9b,d, respectively. The smooth gray areas correspond to the regions with good statistics, where plenty of food particles are accumulated efficiently by the rotation of the setae and quickly move into the mouth. The black spots on the left and right sides of the mouth show places where particles accumulate with time, but cannot enter the mouth and quasi-periodically oscillate over a long period of time. The difference in accumulation for these two cases is obvious and highlights that a system containing both setae types (Figure 9b,d) is optimal for gathering particles from the surrounding water.

The long setae rather generate water currents that bring food particles to the short setae, which contact and capture them [14–18]. The interaction between setae and particles, which depends on setae morphology, mesh size of the filtering structure, and the surface chemistry and forces of feeding structures and particles [14,29–36], has been observed previously under binocular microscopes [19–28]. It has also been determined previously that mechanical property gradients of the setae, with soft bases or soft tips, are important [25,37–38]. Our model clearly depicts that the mechanical properties of the setae, including the adhesiveness of the tip, determine the feeding efficiency. Additionally, it shows that short and long setae are more effective when they work in concert and have different mechanical properties and different adhesiveness.

This present study is rather a protocol for carrying out more extensive numerical modeling in the future as the model can be easily computed with MatLab and the parameters of the model (e.g., the size of the food particles and the quantity and mechanical properties of setae) can be adjusted to a specific system or animal.

## Conclusion

We present here the first numerical model of the feeding setae of crustaceans taking into account the actual physical processes of the environment. The model estimates the particle collecting efficiency depending on mechanical property gradients and adhesion of the different setae. Following this protocol, the model can be easily extended through adjustment of the parameters to fit a specific suspension feeding system or different food items. It also could serve as an inspiration to develop new filtering techniques with adhesive elements retaining particles on the micro- to millimeter scale.

## Experimental

### Specimens studied

As a model organism, we chose *Centropages hamatus* (Crustacea, Copepoda, Calanoida). The mechanical properties of the setae were previously documented by CLSM [55–57]. The short setae on maxilla 2 possess soft tips and soft bases, and the long setae on maxilla 1 possess only soft bases (Figure 1).

### Mathematical model

**Figure 10:**
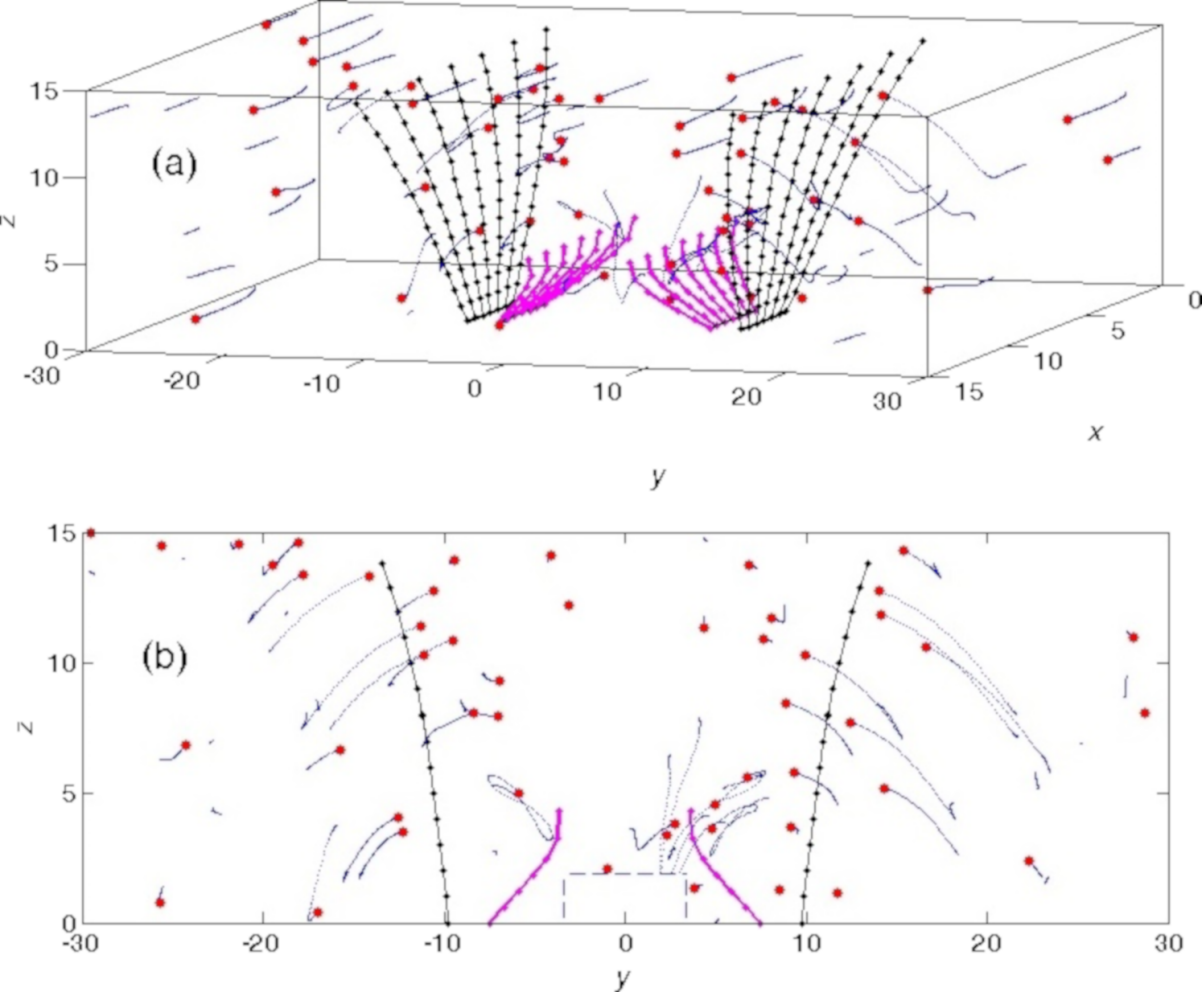
Concept of the numerical model. Setae, arranged as two pairs of seta rows (internal short setae and external long setae), were simulated by lines with small circles. Each small circle separated two elastically connected seta segments. The big red circles represent the instant position of the movable particles (“food”). The dotted comet tails behind each particle visualize small fragments of particle trajectories. (a) shows a three-dimensional view on the system and (b) its projection on the (*y*,*z*) plane. The mouth opening was simulated as the dashed-lined box.

For the simulations, we employed MatLab R2022a (The MathWorks, Inc., Natick, Massachusetts, USA). Our discrete numerical model describes the dynamics of two pairs of initially parallel aligned elastic crests, resembling the maxillae 1 and 2. The conceptual structure of the model is depicted in Figure 10. The dynamic behavior of the model can be found in the movie in Supporting Information File 1.

Each seta is constructed of a number of elastic segments (long setae: *N*_x_ = 14, *N*_z_ = 15; short setae: *N*_x2_ = 14, *N*_z2_ = 7) each having the same length d*R*. The model does not reproduce exactly realistic numbers of the setae segments or particles. It only provides more or less natural dynamics of the process, which allows us to make some conclusions about the efficiency of the feeding process at different properties of the setae.

The segments were provided with longitudinal (*K*^∥^) and transverse (*K*^⟂^) stiffness, *K*^∥^ = *K*^⟂^. The transverse stiffness tends to hold the angle between the neighboring segments close to 180°. According to the goals of this study, we varied the stiffness from segment to segment depending on the hypothetical particular structure.

A deformation of the setae produced elastic forces proportional to the seta stiffness. The forces were described by the following equations (see [62] for the formula):


[1]

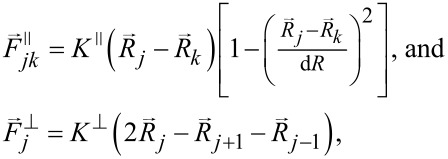



where 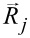 is a position vector of the middle of the segment (the node) *j*; *k* = *j* ± 1. The longitudinal force, 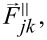 is described here by a double-well potential, which tends to keep the distance between the nodes 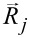 and 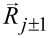 close to the equilibrium length of each segment d*R*.

This particular form of the longitudinal force equation was chosen, because it is linear at small displacement and increases non-linearly at large displacement. The transverse force, 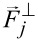, is directly proportional to the lateral deflection and tends to keep the position 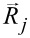 close to the mean value between its nearest neighbors, 
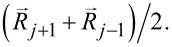
 Additionally, it keeps the direction of every segment as close to parallel with the adjacent ones as possible, at current balance of all forces. The transverse force in the present form is easy to realize numerically, but it is not purely the bending force since this may include a longitudinal component.

In the model, each long seta is constructed from 15 segments and each short one from seven segments. Each seta is rotated around a base segment from minimal to maximal angles, ϕ_min_ and ϕ_max_, respectively. We varied these angles in a wide interval to simulate the different rotational mobility of the individual long and short setae. The angular velocity (frequency of the rotation in both directions) was also widely varied for each seta.

The food was represented by an array of *N*_p_ = 50 particles moving in three-dimensional space with periodic boundary conditions. The particles were created following established protocols [63–68]. For all results presented below, the number *N*_p_ = 50 was fixed as a compromise between a statistically representative value and the computational cost of the calculations.

It was supposed that every particle interacts viscously with a “water flow” caused by both external flow and motion of the setae. Instead of solving time-consuming Stokes equations (which in such a case should involve also a motion of the food particles and movable segments of the setae, making a general solution of the problem extremely complex), we simulate a presence of the liquid by a “mutual friction” between the particles and setae, which aims to reduce the difference between the particle and setae velocity at relatively short distances according to simple factors in the dynamic equations of motion. The particles move in an “empty space”, but interact with the flows caused by the eternal flux and the motion of the setae via an “imaginary liquid”, which influences their motion.

Initially, the particles were placed randomly in a box [0,*L*_x_; −*L*_y_,*L*_y_; 0,*L*_z_] and their velocity was equal to the velocity of external “water flow”, *v*_ext_. The *x* component of the external velocity is *v*_ext_ = 1.2, and the two other components are zero. We chose, for simplicity, a constant flow along one of the coordinates with a relatively low velocity compared to the local instant velocities caused by the motion of the setae. This flow transported the food particles to the region where all further handling of the particles took place. This allowed for the permanent generation of particles outside the system to substitute the eaten particles.

If any particle leaves the box, it is randomly injected back to the system with the same velocity. The same is also done in the case of the particle being “eaten”. A particle is treated as “eaten” when it appears inside the region “mouth”, which is represented by a small box in the center of the ground plane with rectangular (parallelepipedal) borders: [0,*L*_mouth,x_; −*L*_mouth,y_,*L*_mouth,y_; 0,*L*_mouth,z_].

Being separated by water, the particles move practically independently of one another. Thus, particles do not interact directly with each other in our model. However, each particle interacts with the setae via the liquid. Due to strong damping, each particle tends to equilibrate its speed with the local velocity of the liquid. This velocity, in turn, is determined by a combination of the external flow *v*_ext_ and perturbations caused by the motion of the setae.

For simplicity and to increase calculation efficiency, instead of solving the huge complete set of dynamic and hydrodynamic equations for particles of complex form with rough surfaces, we separated the potential (exponentially depending on the distance) interaction (interaction caused by a mutual friction, which tends to reduce a difference between velocities).

The appropriate force from the setae acting on every particle is represented as a combination of the following velocity- and distance-dependent factors (see [62] for the formula):


[2]

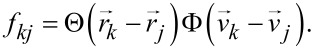



Here, the first factor describes the tendency to equilibrate the velocities of every particle and each segment of the seta. The second one determines how this interaction decays with the distance between the particle and segment. As a first approximation, one can accept that these factors linearly depend on the difference between the velocities and exponentially decrease with the distance in phase (velocity, coordinate) space {*v*,*r*} between the chosen particle and each segment:


[3]

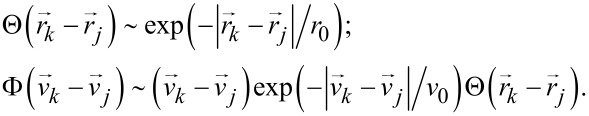



As it was mentioned, the particles are involved in the water motion. Thus, the same velocity-dependent interaction exists between the particles and the external flow of water. It can be written in the form


[4]

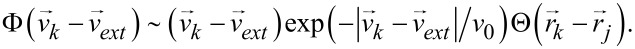



Besides the equilibration of the velocities, there is a direct mechanical (or chemical) interaction between the setae and the particles. This interaction becomes important especially for the thin elastic ends of the short setae near the mouth opening. In particular, the adhesion by van der Waals attraction becomes possible at such scales. This part of the interaction must also be included in the model, namely in a form of potential interaction between the setae (or their tips) and the food particles. The corresponding force can be written in the following gradient form:


[5]

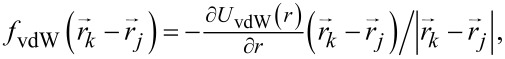



where 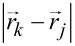 is the modulus of the difference, and 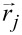 is the position of the particle. For definiteness and minimization of the numerical calculations *U*_vdW_(*r*) can be represented by a relatively simple Morse potential *U*_vdW_(*r*) = *U*_0_(1 – exp(−*a*(*r* – *r*_vdW_)))^2^, where *r*_vdW_ is the position of the minimum of the van der Waals potential.

The combined influence of all forces mentioned above leads to a typical dynamic scenario, which has been recorded in the movie in Supporting Information File 1. It reproduces quite realistically the behavior of particles moving around a real animal [69].

It is important to note that due to the randomness of the initial conditions and the injection of “eaten” particles back into the system, particular feeding sequences never exactly repeat. However, after a short transient period, a well-defined quasi-periodic (“strange attractor”) motion self-organized in the system, which could be easily analyzed statistically. Besides, one can vary the parameters of the model equation and obtain similar kinds of behavior.

## Supporting Information

File 1 Dynamic behavior of the model.

## References

[1] Jørgensen C B (1966). Biology of suspension feeding.

[2] Jørgensen C B (1975). Annu Rev Physiol.

[3] Strathmann R R, Giese A C, Pearse J S, Pearse V B (1987). Larval feeding. Reproduction of marine invertebrates. Vol. IX. General aspects: seeking unity in diversity.

[4] Wotton R S, Wotton R S (1994). Methods for capturing particles in benthic animals. The biology of particles in aquatic systems.

[5] Riisgård H U, Larsen P S (1995). Biol Rev Cambridge Philos Soc.

[6] Riisgård H U, Larsen P S (2000). Limnol Oceanogr.

[7] Riisgård H U, Larsen P S (2001). Limnol Oceanogr.

[8] Hentschel B T, Shimeta J, Jørgensen S E, Fath B D (2008). Suspension feeders. Encyclopedia of Ecology.

[9] Riisgård H U, Larsen P S (2010). Mar Ecol: Prog Ser.

[10] Kiørboe T (2011). Biol Rev.

[11] Hamann L, Blanke A (2022). J R Soc, Interface.

[12] Garm A, Watling L, Thiel M, Watling L (2013). The crustacean integument: setae, setules, and other ornamentation. The Natural History of the Crustacea.

[13] Riisgård H U, Thiel M, Watling L (2015). Filter-feeding mechanisms in crustaceans. Lifestyles and feeding biology. The Natural History of the Crustacea.

[14] Rubenstein D I, Koehl M A R (1977). Am Nat.

[15] LaBarbera M (1984). Am Zool.

[16] Jumars P A (1993). Concepts in biological oceanography. An interdisciplinary primer;.

[17] Vogel S (1994). Life in moving fluids. The physical biology of flow.

[18] Shimeta J, Koehl M A R (1997). J Exp Mar Biol Ecol.

[19] Rosenberg G G (1980). Limnol Oceanogr.

[20] Paffenhöfer G-A, Strickler J R, Alcaraz M (1982). Mar Biol (Heidelberg, Ger).

[21] Wägele J W (1987). Philos Trans R Soc, B.

[22] Gerritsen J, Porter K G, Strickler J R (1988). Bull Mar Sci.

[23] Hamner W M, Hamner P P (2000). Can J Fish Aquat Sci.

[24] Garm A, Høeg J T (2001). Biol Bull (Chicago, IL, U S).

[25] Garm A, Hallberg E, Høeg J T (2003). Biol Bull (Chicago, IL, U S).

[26] Garm A (2004). J Morphol.

[27] Gonçalves R J, van Someren Gréve H, Couespel D, Kiørboe T (2014). Mar Ecol: Prog Ser.

[28] Giuffre C, Hinow P, Jiang H, Strickler J R (2019). Sci Rep.

[29] Koehl M A R, Strickier J R (1981). Limnol Oceanogr.

[30] Gerritsen J, Porter K G (1982). Science.

[31] Gophen M, Geller W (1984). Oecologia.

[32] Ganf G G, Shiel R J (1985). Aust J Mar Freshwater Res.

[33] Monger B C, Landry M R (1990). Mar Ecol: Prog Ser.

[34] Garm A, Høeg J T (2000). Mar Biol (Heidelberg, Ger).

[35] Koehl M A R (2004). J Biomech.

[36] Geierman C, Emlet R (2009). J Exp Mar Biol Ecol.

[37] Garm A (2005). Mar Biol (Heidelberg, Ger).

[38] Vittori M, Srot V, Bussmann B, Predel F, van Aken P A, Štrus J (2018). Micron.

[39] Visser A W (2001). Mar Ecol: Prog Ser.

[40] Yen J, Okubo A (2002). Hydrobiologia.

[41] Shen X, Yao X, Marcos, Fu H C (2021). Limnol Oceanogr.

[42] Cannon H G (1928). Br J Exp Biol.

[43] Marshall S M, Orr A P (1966). J Mar Biol Assoc U K.

[44] Boyd C M (1976). Limnol Oceanogr.

[45] Nival P, Nival S (1976). Limnol Oceanogr.

[46] Nival P, Nival S (1979). Limnol Oceanogr.

[47] Price H J, Paffenhöfer G-A (1986). Limnol Oceanogr.

[48] Hansen B, Tiselius P (1992). J Plankton Res.

[49] Kiørboe T, Saiz E (1995). Mar Ecol: Prog Ser.

[50] Kiørboe T (1997). Sci Mar.

[51] Kiørboe T (2008). A mechanistic approach to plankton ecology.

[52] Jansen S (2006). Feeding behaviour of calanoid copepods and analyses of their faecal pellets.

[53] Tiselius P, Saiz E, Kiørboe T (2013). Limnol Oceanogr.

[54] Kadiene E U, Ouddane B, Hwang J-S, Souissi S (2019). Sci Rep.

[55] Michels J, Gorb S N (2012). J Microsc (Oxford, U K).

[56] Michels J, Gorb S N (2015). Beilstein J Nanotechnol.

[57] Michels J, Reynaud E G (2013). Confocal laser scanning microscopy – detailed three-dimensional morphological imaging of marine organisms. Imaging marine life: macrophotography and microscopy approaches for marine biology.

[58] Peisker H, Michels J, Gorb S N (2013). Nat Commun.

[59] Gorb S N (2005). Am Entomol.

[60] Gorb S N (2008). Philos Trans R Soc, A.

[61] Büscher T H, Gorb S N (2021). Beilstein J Nanotechnol.

[62] Filippov A E, Gorb S N (2020). Combined Discrete and Continual Approaches in Biological Modelling.

[63] Monaghan J J (1988). Comput Phys Commun.

[64] Pöschel T, Schwager T (2005). Computational granular dynamics: Models and algorithms.

[65] Hoover W G (2006). Smooth particle applied mechanics. The state of the art.

[66] Radjai F, Dubois F (2011). Discrete-element modeling of granular materials.

[67] Dmitriev A I, Nikonov A Y, Filippov A E, Psakhie S G (2019). Phys Mesomech.

[68] Filippov A E, Popov V L (2023). Facta Univ, Ser: Mech Eng.

[69] (2023). Youtube, Strickler Lab.

